# Supplementary material for: Sustained micellar delivery via inducible transitions in nanostructure morphology
Source: Nat Commun. 2018 Feb 12;9:624. doi: 10.1038/s41467-018-03001-9 (PMC5809489; doi:10.1038/s41467-018-03001-9)
Supplement: Supplementary file 1 — Supplementary Information [file 41467_2018_3001_MOESM1_ESM.pdf]

Sustained Micellar Delivery via Inducible Transitions in Nanostructure Morphology

Karabin et al.

Supplementary Information

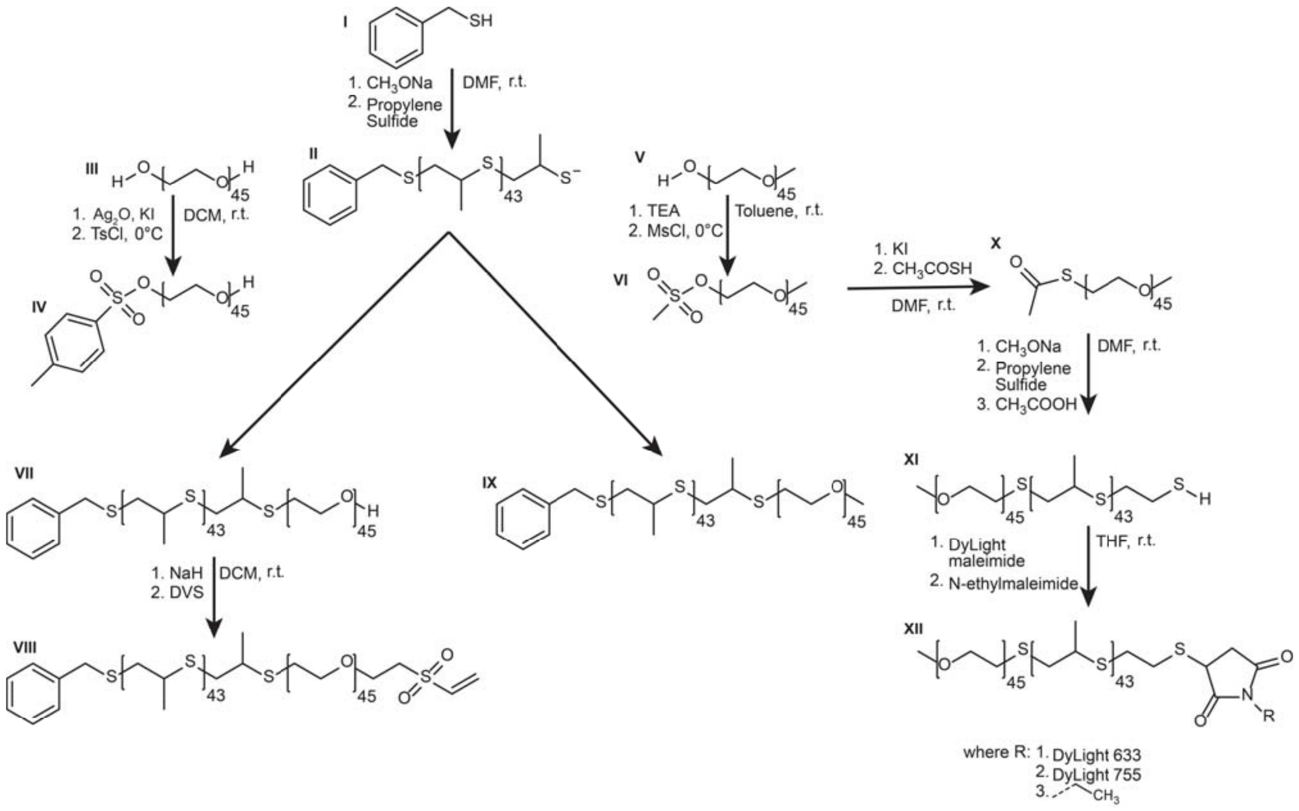

Supplementary Figure 1 Complete synthetic schema for PEG-bl-PPS polymers used in this study. A small molecule thiol-containing initiator, in this case benzyl mercaptan (**I**) is 1. activated with base and 2. used to perform ring-opening polymerization on propylene sulfide to produce poly(propylene sulfide) homopolymer (**II**). To be used as an end capping agent for **II**, hydroxyl PEG (**III**) or methyl ether PEG (**V**) must first be functionalized with a leaving group, such as a tosylate or mesylate group to form hydroxyl PEG monotosylate (**IV**) or methyl ether PEG mesylate (**VI**). These two PEG derivates are used to end cap **II**, resulting in products **VII** and **IX**, respectively. The available hydroxyl on **VII** is subsequently converted into a vinyl sulfone group to form VS-PEG-bl-PPS (**VIII**). To synthesize the DyLight-conjugated polymer, methyl ether PEG mesylate (**VI**) must first be converted into a thiol-containing compound, through reaction with thioacetic acid, to form methyl ether PEG thioacetate (**X**). This is reacted in the same manner as the synthesis of compound **II**, to form PEG-bl-PPS-Thiol (**XI**). This thiol group is available for covalent conjugation with maleimide groups to afford a number of conjugated PEG-bl-PPS compounds (**XII**).

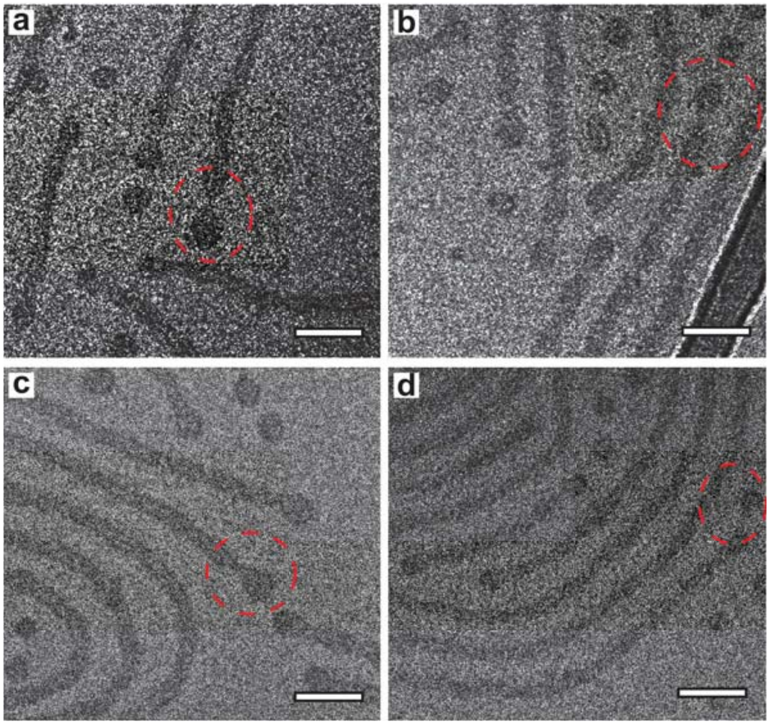

Supplementary Figure 2. Additional cryoTEM images of micellar budding from filomicelles. **a-c**. Cryogenic transmission electron micrographs of micellar budding from PEG-bl-PPS filomicelles and **d.** DyLight conjugated PEG-bl-PPS filomicelles, with dotted red circles highlighting examples of budding. All images are at the same magnification, all scale bars represent 50 nm.

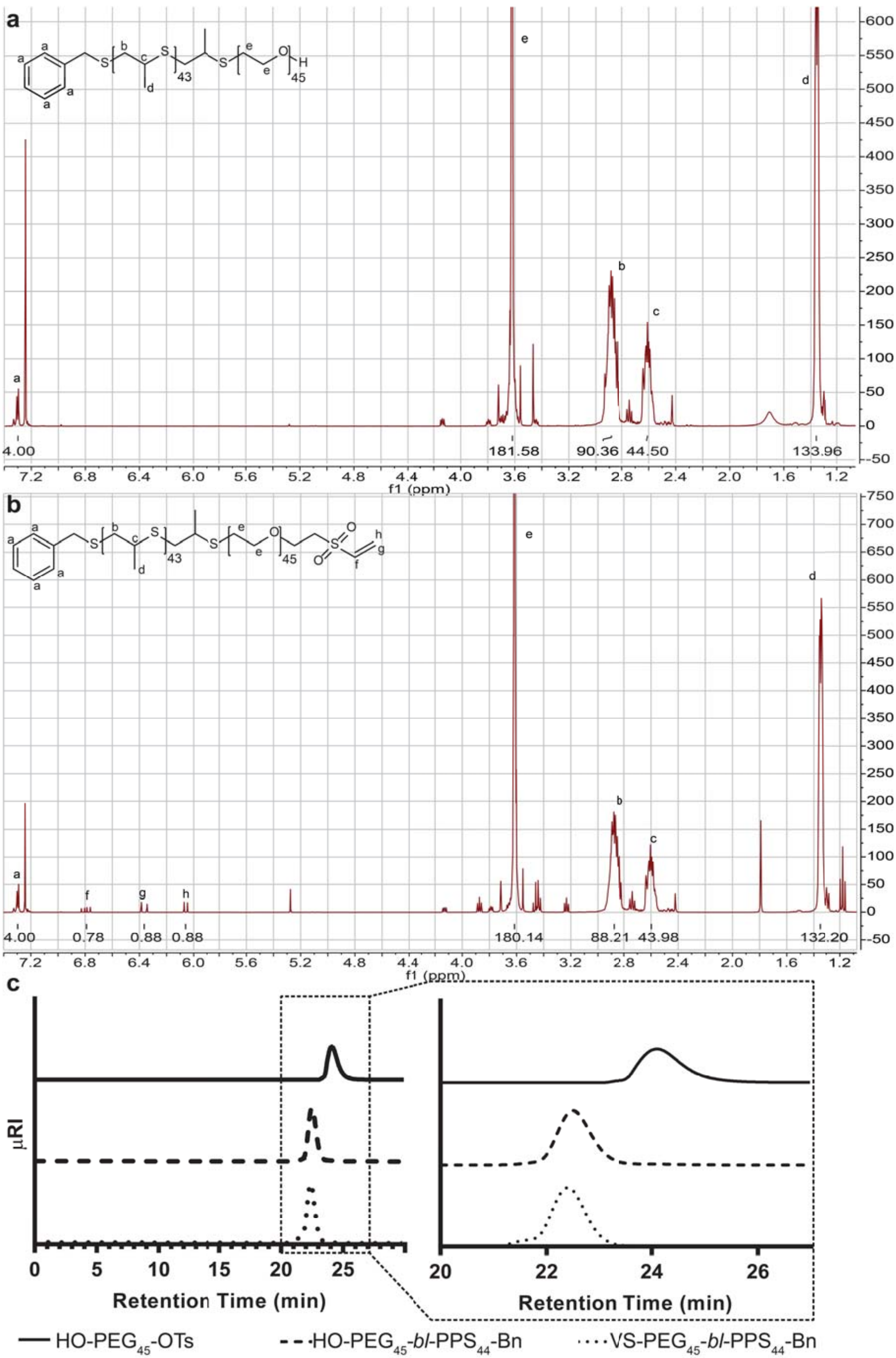

a
b
c
f1 (ppm)
µRI
Retention Time (min)
HO-PEG45-OTs
HO-PEG45-bl-PPS44-Bn
VS-PEG45-bl-PPS44-Bn

Supplementary Figure 3. Characterization of PEG_{45}-bl-PPS_{44} polymers. **a.** ^{1}H NMR spectra of HO-PEG_{45}-bl-PPS_{44}. **b.** ^{1}H NMR spectra of VS-functionalized PEG_{45}-bl-PPS_{44}. **c.** Overlay of gel permeation chromatography chromatograms for polymer precursors of VS-functionalized PEG_{45}-bl-PPS_{44}.

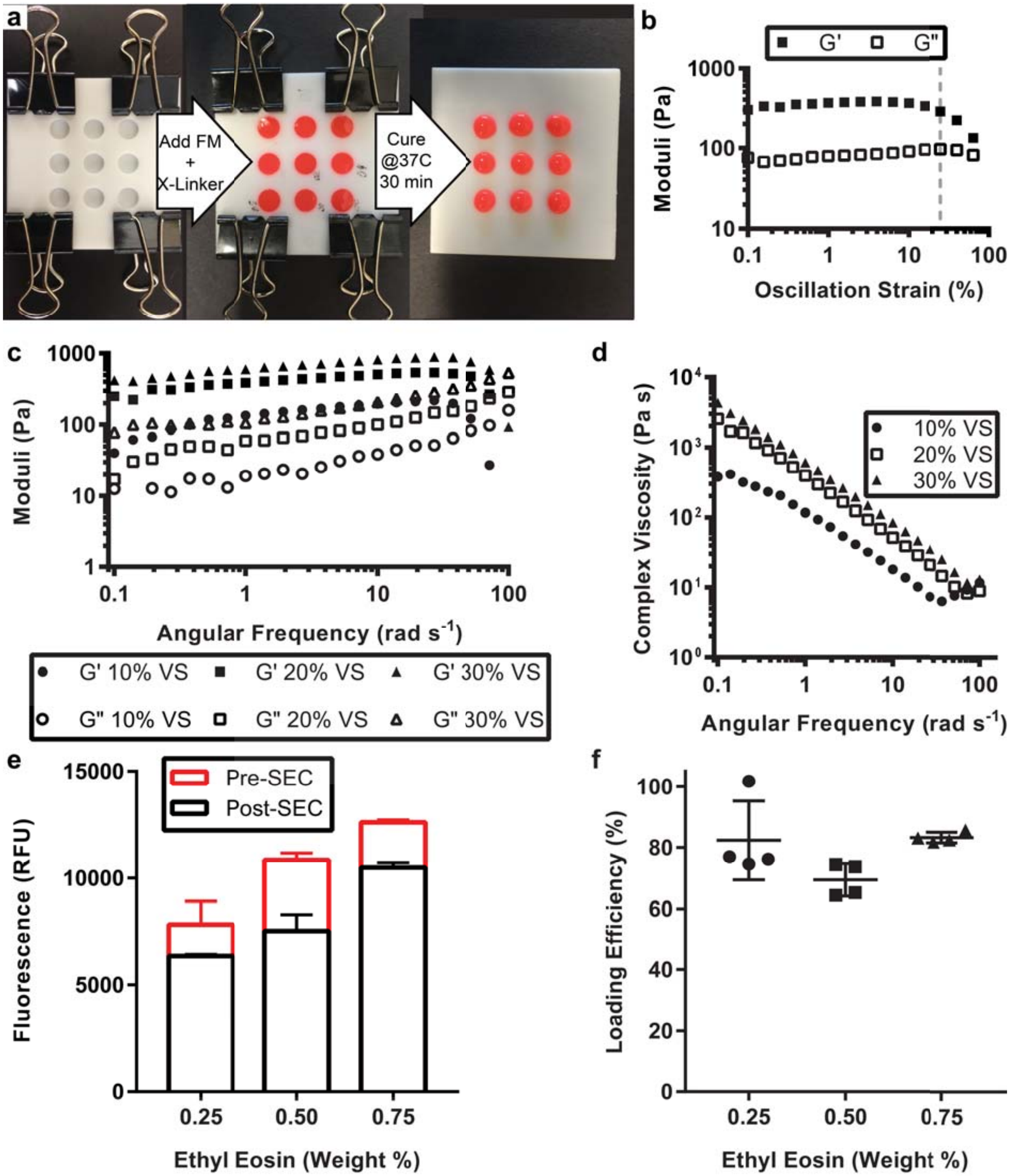

Supplementary Figure 4. Ex vivo formation and characterization of FM-scaffolds. **a.** Images depicting the formation of FM-scaffolds for in vitro studies. Teflon molds were filled with a 10 wt% solution of ethyl eosin-loaded FM along with corresponding volumes of the 8-arm PEG-SH crosslinker. Plated FM solutions were cured for 30 minutes at 37°C in a humidified environment to produce the FM-scaffolds. **b.** Strain-dependent oscillatory rheology of FM-scaffolds (10 wt%) incorporating 20% VS-BCP **(**Angular frequency = 6.28 rad s^{-1}, 37°C). Frequency-dependent oscillatory rheology of **c.** storage and loss moduli and **d.** complex viscosities for FM-scaffolds (10 wt%) exhibiting varying percentages of VS-BCP depicting (Strain = 0.5%, 37°C). **e.** Fluorescence intensity readings of loaded ethyl eosin within FM before and after gravity size exclusion on a sepharose 6B column and **f.** loading efficiency derived from a single purification with four measurement replicates, n=4, s.d.

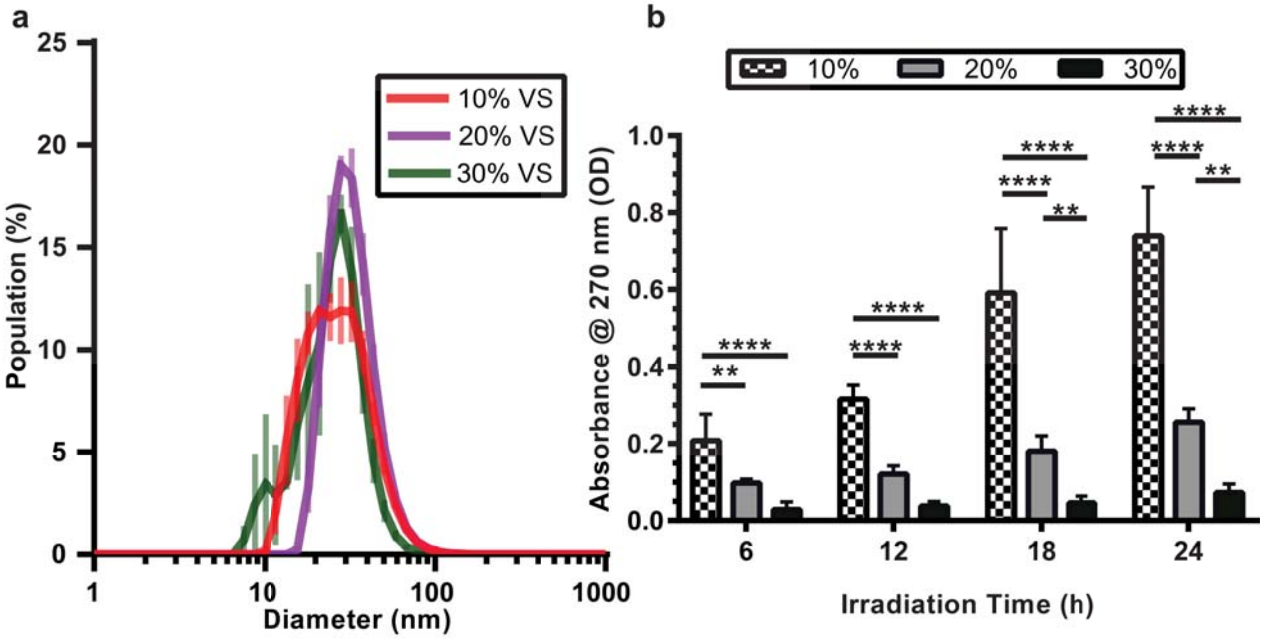

Supplementary Figure 5. Photo-oxidation of PEG-bl-PPS FM-scaffolds increases release of monodisperse micelles with time. **a.** DLS size distribution of nanocarriers released from scaffolds irradiated for 24 h. Error bars represent SD, n=3 for all groups. **b.** Absorbance at 270 nm of released PEG-bl-PPS in supernatant following scaffold irradiation for 6, 12, 18, and 24 h. Significance between groups assessed by 2-way RM ANOVA with Tukey's multiple comparison, * $p < 0.05$, ** $p < 0.01$, *** $p < 0.001$, and **** $p < 0.0001$. Error bars represent SD, n=7 for all groups except scaffolds incorporating 10% VS-BCP and 20% VS-BCP irradiated for 18 h where n=6.

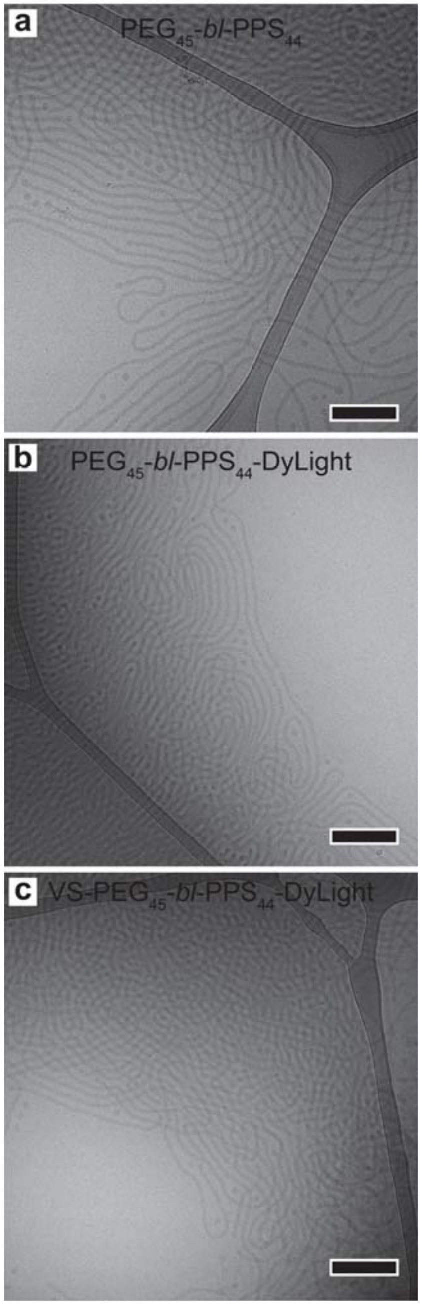

Supplementary Figure 6. CryoTEM confirmation of FM morphology for DyLight incorporating structures. CryoTEM micrographs comparing FMs prepared from **a.** MeO-BCP, **b.** MeO-BCP, 633-BCP, and 755-BCP, and **c.** MeO-BCP, VS-BCP, 633-BCP, and 755-BCP. Scale bar, 200 nm.

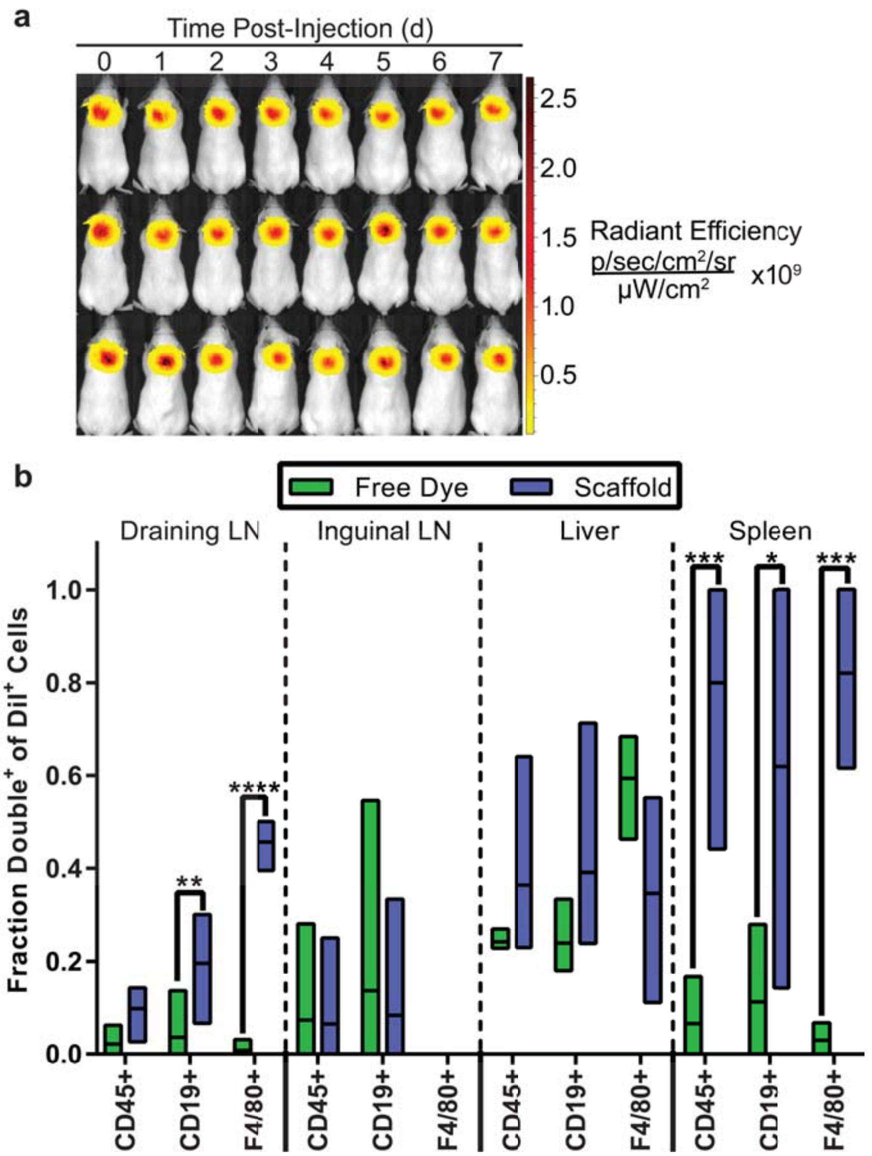

Supplementary Figure 7. IVIS images and additional flow cytometric analysis for colocalization study. IVIS images for all mice injected with DyLight 755-BCP modules within 4-component FMs of in situ crosslinked scaffolds which were additionally loaded with lipophilic dye DiI. b, Additional flow cytometric analysis of DiI and 633-BCP uptake by immune cell populations in vivo. The y-axis represents the fraction of DiI+ cells that were also positive for 633-BCP. Floating columns represent min, mean, and max, n=4. Significance was determined by Mann-Whitney U Test, two-tailed. * $p<0.05$, ** $p<0.01$, *** $p<0.001$, **** $p<0.0001$.

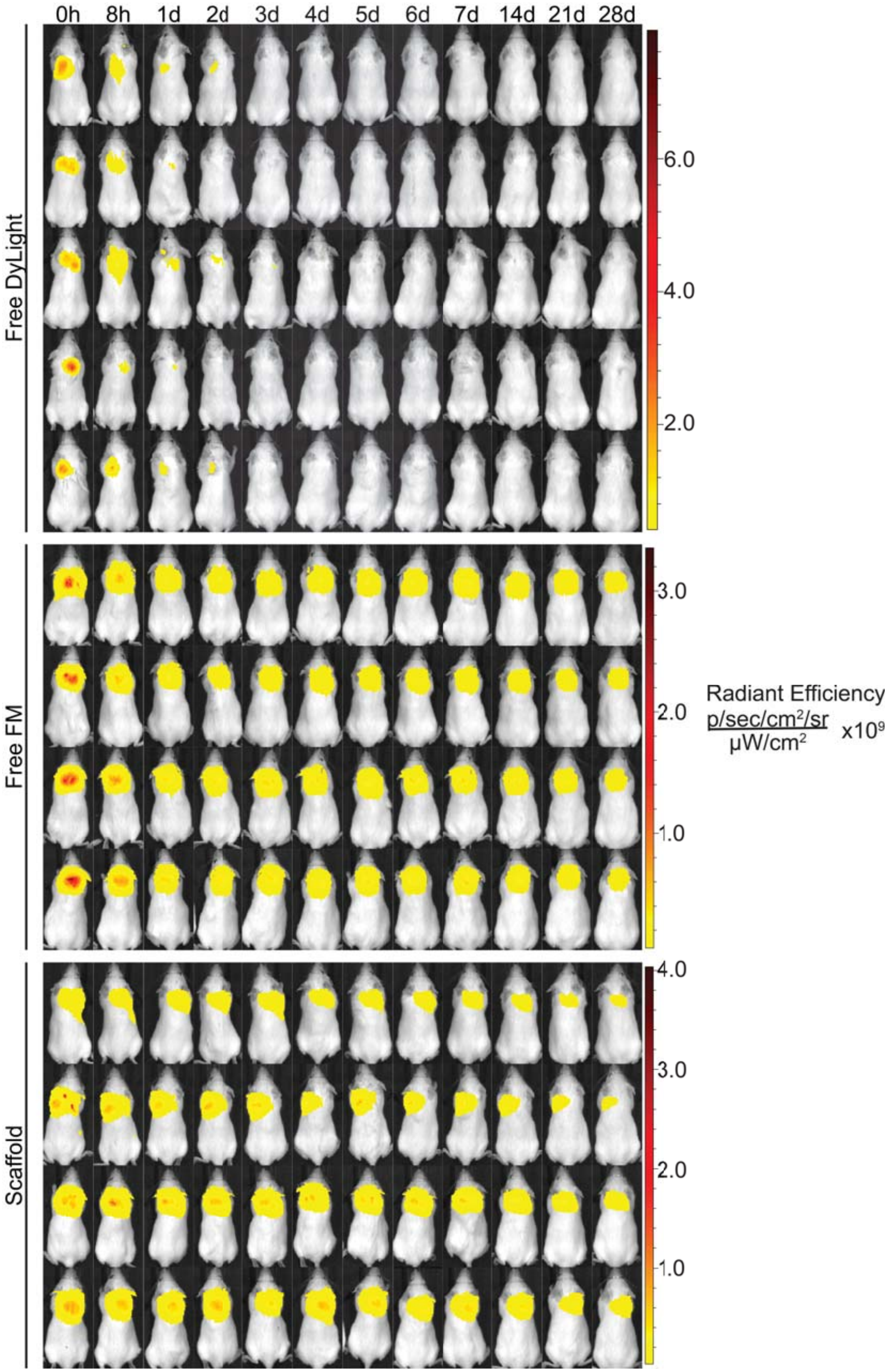

Supplementary Figure 8. Complete IVIS image set for 28-day study. IVIS images for all mice injected with Dylight 755 as: free solubilized dye, conjugated to 755-BCP modules within uncrosslinked 4-component modular FMs, or conjugated to 755-BCP modules within 4-component FMs of in situ crosslinked scaffolds. Radiant efficiency scaled per injection group.

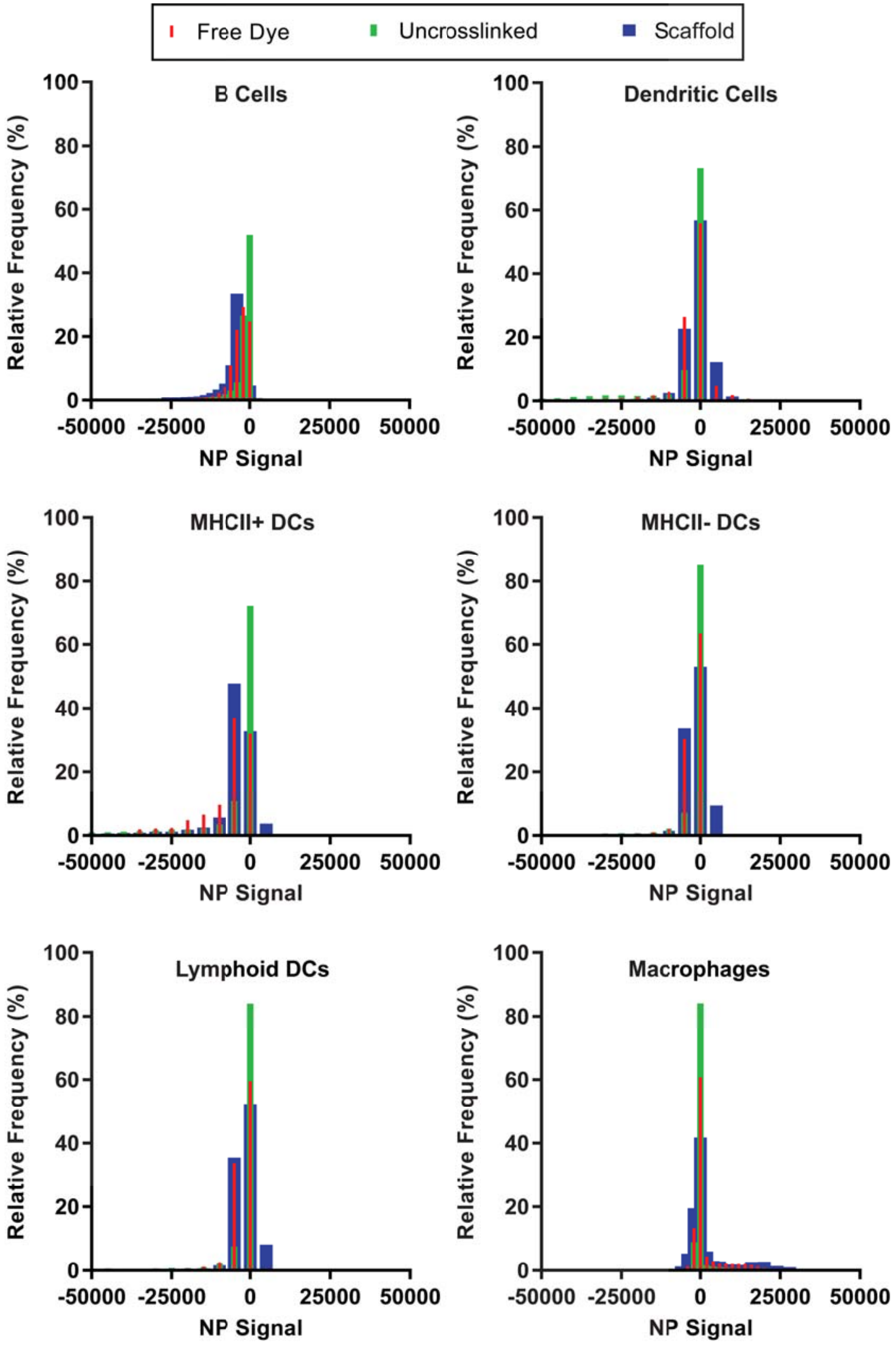

Supplementary Figure 9. Additional flow cytometric analysis for in vivo degradation studies. Histograms of median fluorescence intensity of DyLight 633 signal within the draining lymph node. MFI calculated after compensation, which leads to the presence of some cells exhibiting negative MFI values. N = 5 mice for free dye and n=4 mice for uncrosslinked and scaffold groups.

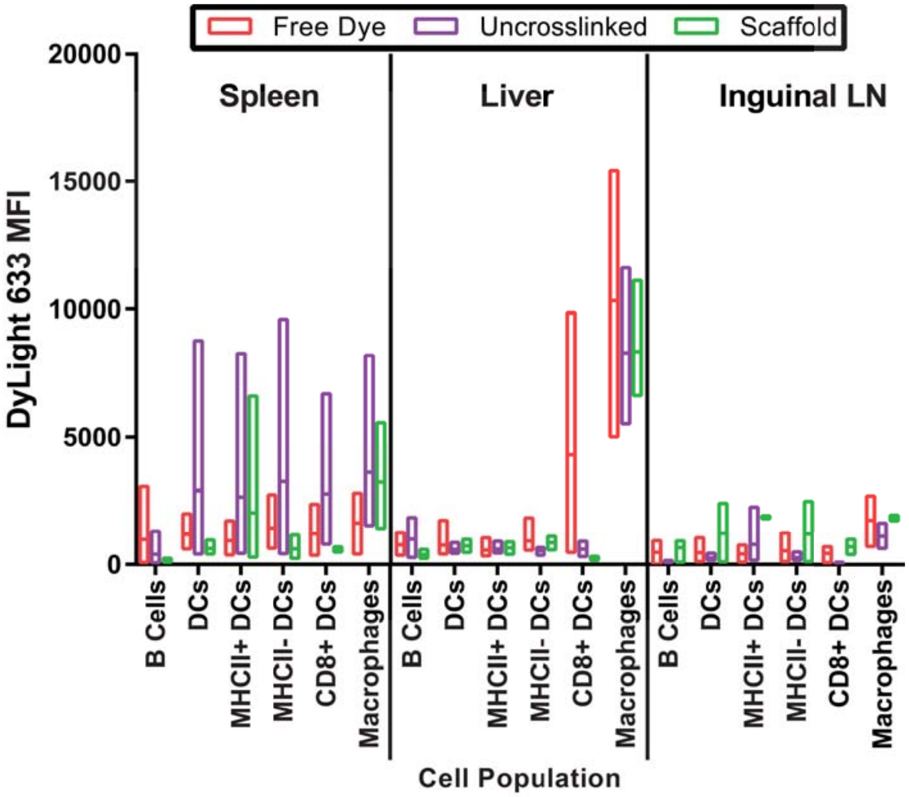

Supplementary Figure 10. Additional flow cytometric analysis for in vivo degradation studies. Additional flow cytometric analysis of 633-BCP uptake by immune cell populations in vivo, 28 days post-injection. Floating columns represent min, mean, and max, n=5 mice for free dye and n=4 mice for uncrosslinked and scaffold groups.

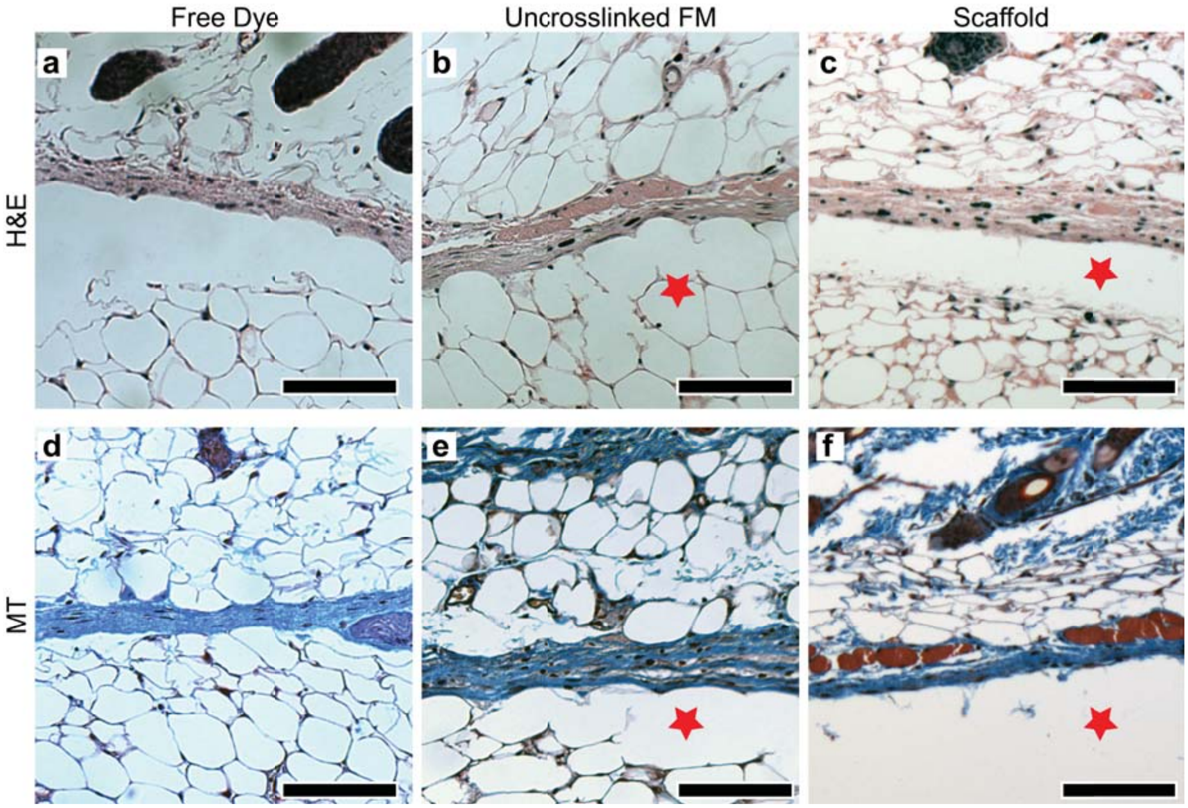

Supplementary Figure 11. Histological analysis of tissue at the injection interface. **a-c**. Representative images of Masson's Trichrome staining of the interface between skin and the saline Dylight 633 and DyLight 755 solution-injected control, uncrosslinked FM, or the in situ crosslinked FM-scaffold, respectively. **d-f**, Representative images of H&E staining of the interface between skin and the saline Dylight 633 and DyLight 755 solution-injected control, uncrosslinked FM, or the in situ crosslinked FM-scaffold, respectively. All images shown are at 10x objective magnification, scale bars are 100 µm.

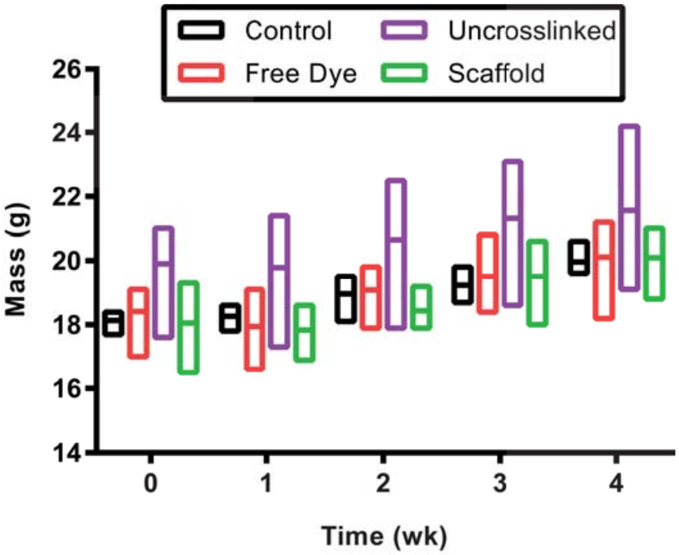

Supplementary Figure 12. Comparison of mouse masses between treatments over the course of in vivo degradation study. Mouse masses were recorded weekly over the course of the in vivo degradation study. Comparison of the DPBS control mice to the free DyLight, uncrosslinked FMs, and in situ crosslinked scaffold treated mice revealed no statistically significant difference in average mouse mass within each time point. Floating columns represent min, mean, and max. n=3 mice for control group, n=5 mice for the free dye group, and n=4 mice for the uncrosslinked and in situ crosslinked scaffold groups.

One Week DiI 633-BCP Co-Localization Panel

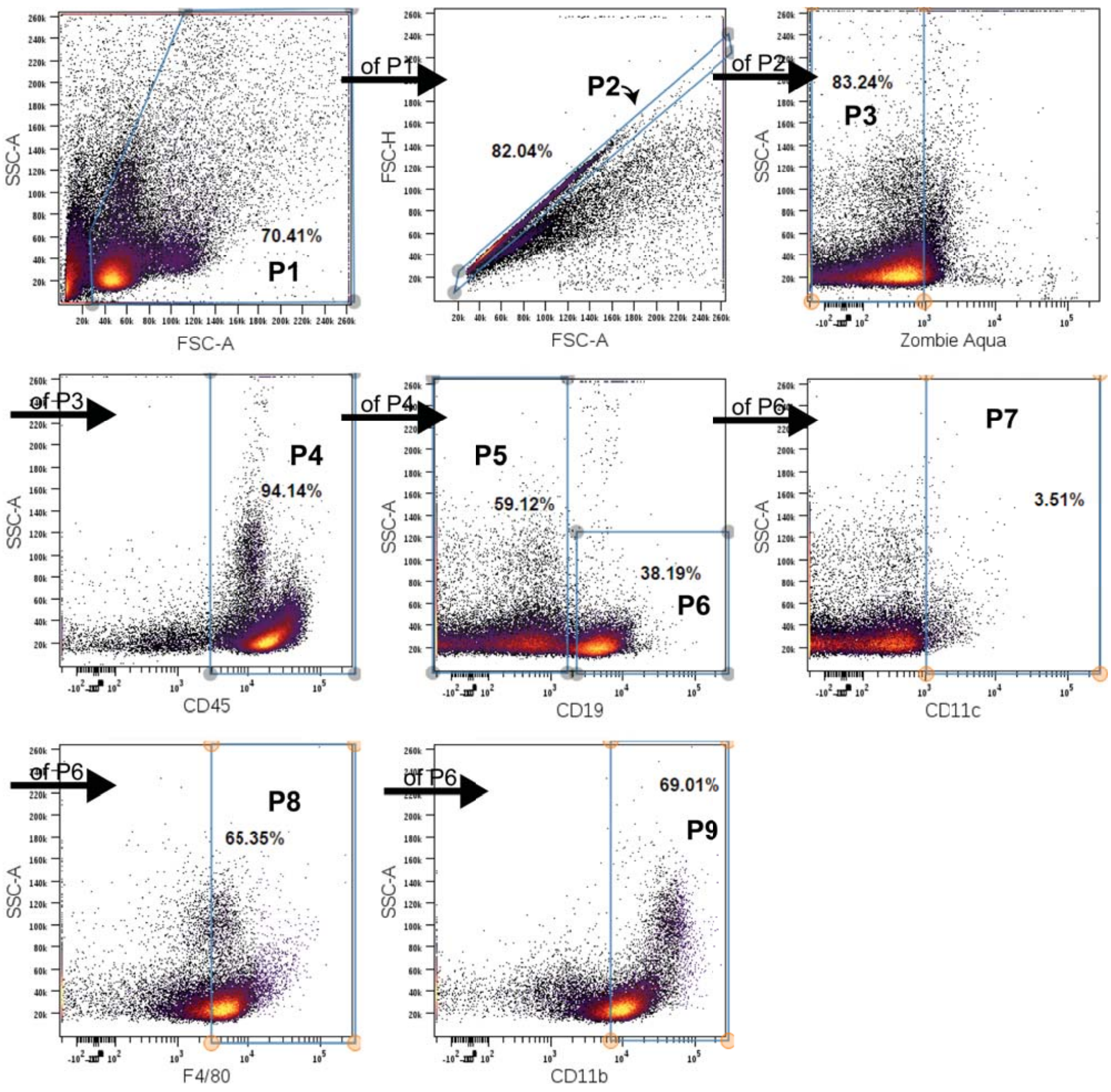

28 Day Post-Injection 633-BCP Biodistribution Panels

Panel 1

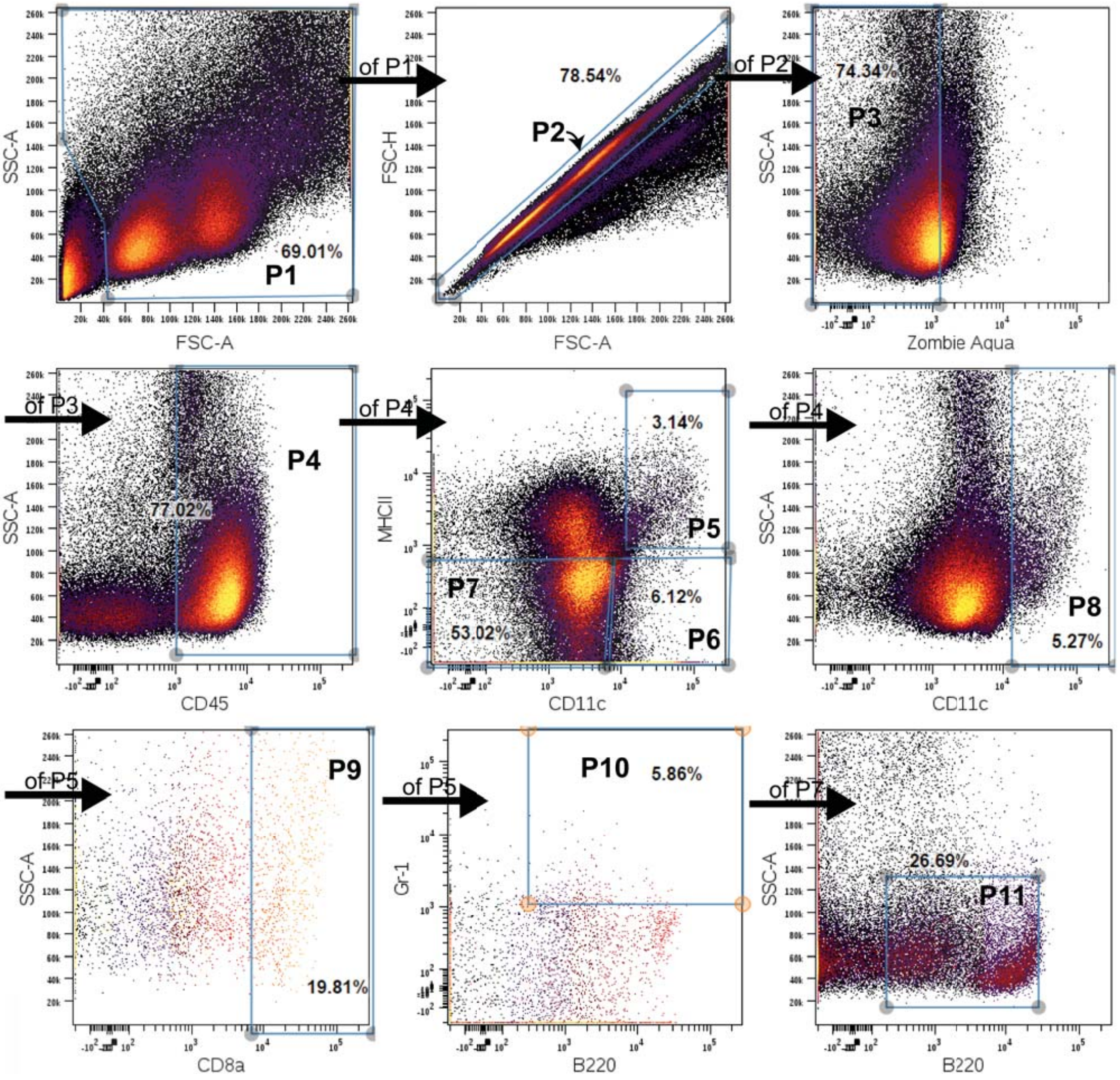

Panel 2

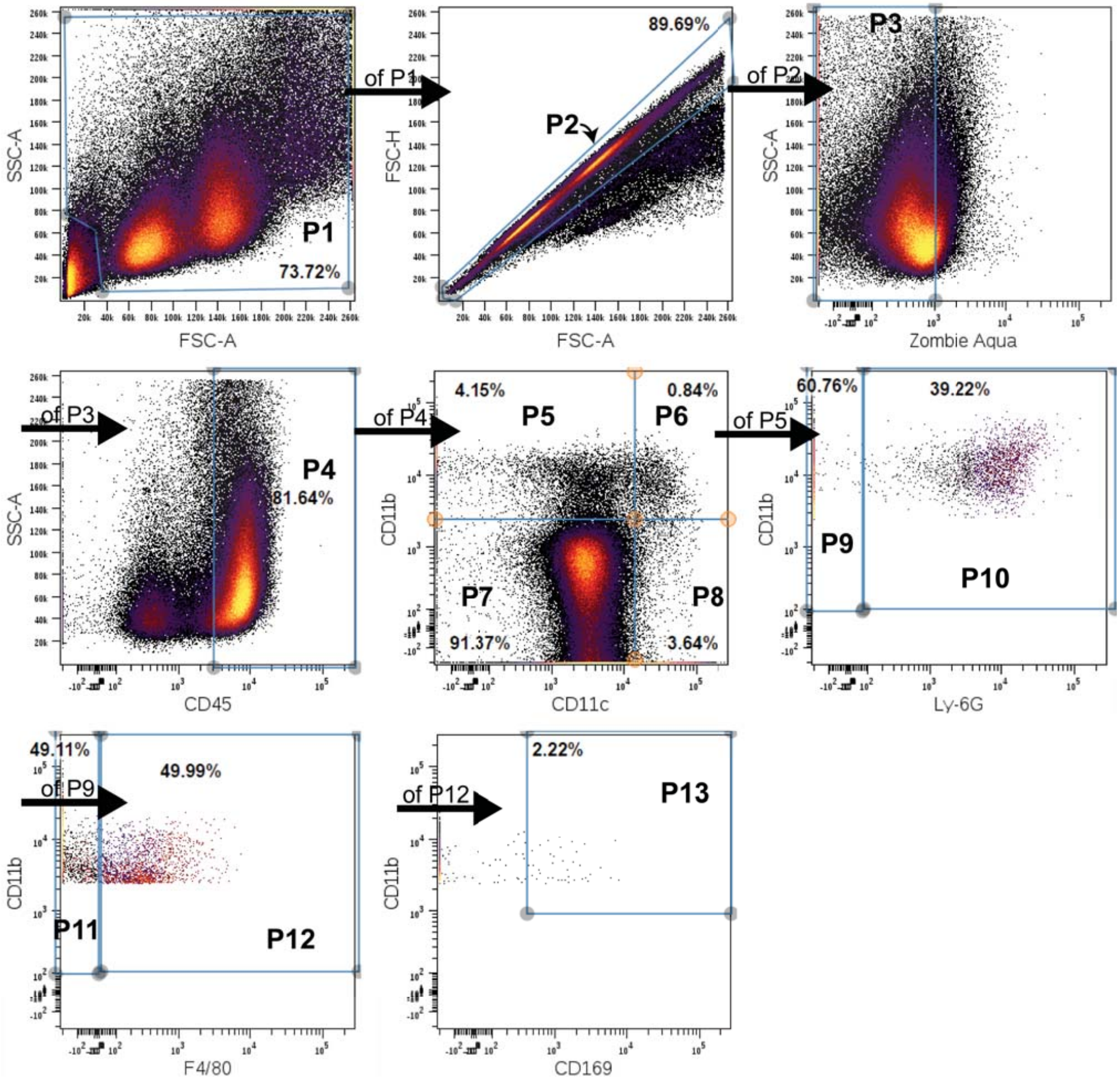

Supplementary Figure 13. Gating strategies for flow cytometry studies. Part 1 - One Week DiI 633-BCP Co-Localization Panel: Gating strategy for the one week DiI 633-BCP co-localization experiment. P1: non-debris events, P2: singlets, P3: living cells, P4: CD45+ cells, P5: non-B cells, P6: B cells, P7: CD11c+ cells (dendritic cells), P8: F4/80+ cells (macrophages), P9: CD11b+ cells (myeloid cells: monocytes and some granulocytes). Parts 2 and 3 - 28 Day Post-Injection 633-BCP Biodistribution Panels: Gating strategy for the 28 day post-injection 633-BCP biodistribution experiment. Panel 1 – P1: non-debris events, P2: singlets, P3: living cells, P4: CD45+ cells, P5: CD11c+ MHCII+ (mature dendritic cells), P6: CD11c+ MHCII- cells (immature dendritic cells), P7: CD11c- MHCII- cells (non-dendritic cells), P8: CD11c+ cells (all dendritic cells), P9: CD8a+ mature dendritic cells (lymphoid dendritic cells), P10: B220+ Gr-1+ mature dendritic cells (plasmacytoid dendritic cells), P11: B220+ non-dendritic cells (B cells). Panel 2 – P1: non-debris events, P2: singlets, P3: living cells, P4: CD45+ cells, P5: CD11b+ CD11c- cells, P6: CD11b+ CD11c+ cells (myeloid dendritic cells), P7: CD11b- CD11c- cells, P8: CD11b- CD11c+ cells (other dendritic cells), P9: CD11b+ Ly-6G- cells, P10: CD11b+ Ly-6G+ cells (neutrophils), P11: CD11b+ F4/80- cells (monocytes), P12: F4/80+ cells (macrophages), P13: CD169+ cells (resident macrophages).
