# Supplementary material for: Sustained micellar delivery via inducible transitions in nanostructure morphology
Source: Nat Commun. 2018 Feb 12;9:624. doi: 10.1038/s41467-018-03001-9 (PMC5809489; doi:10.1038/s41467-018-03001-9)
Supplement: Supplementary file 3 — Description of Additional Supplementary Files [file 41467_2018_3001_MOESM3_ESM.pdf]

Description of Additional Supplementary Files

File Name: Supplementary Movie 1
Description: Three-dimensional cryoTEM tomography of micelle release from PEG-bl-PPS filomicelles.
